# Vaccination uptake among Australian early childhood education staff: assessing perceptions, behaviours and workplace practices

**DOI:** 10.1186/s12879-019-4427-z

**Published:** 2019-09-14

**Authors:** Holly Seale, Stephanie Dwyer, Alamgir Kabir, Rajneesh Kaur

**Affiliations:** 1 School of Public Health and Community Medicine, University of New South Wales, Level 2, Samuels Building, Randwick, Sydney, NSW 2031 Australia; 2 Psychosocial Research Group, Prince of Wales Clinical School, University of New South Wales, Sydney, Australia

**Keywords:** Immunization, Vaccine, Pediatric, Childcare, Influenza, Pertussis

## Abstract

**Background:**

Early Childhood Education Centre (ECEC) staff are strongly recommended to receive several immunizations including influenza and pertussis. However, evidence regarding the uptake is either old or lacking across all Australian States/Territories. This study aimed to explore the attitudes and barriers around ECEC staff vaccination and the immunisation policy/practices employed at their workplaces.

**Methods:**

An online cross-sectional survey was undertaken of staff members (administrators and childcare center staff) in early 2017. We compared the individual’s knowledge, attitude and practices as well as the centre’s policy and practice variables between the vaccinated and unvaccinated respondents. A logistic model was used to identify the factors associated with uptake of the different vaccines.

**Results:**

A total of 575 ECEC staff completed the survey. Sixty percent reported being aware of the recommendations about staff immunisation. While participants did acknowledge that they could spread diseases if unvaccinated (86%), 30% could not recall receiving a dTpa in the last 10 years. Private centres were less likely to provide free or onsite vaccination compared to other categories of centres. Less than half reported receiving any encouragement to get the influenza vaccine and only 33% reported that their centre provides onsite influenza vaccination. Regarding the introduction of mandatory policies, 69% stated that they would support a policy.

**Conclusion:**

Employers should consider supporting methods to maximize vaccination of their employees including providing free onsite vaccination. Participants were open to idea of mandatory vaccination; however, this needs to be explored further to determine how vaccine costs and access issues could be resolved.

**Electronic supplementary material:**

The online version of this article (10.1186/s12879-019-4427-z) contains supplementary material, which is available to authorized users.

## Background

The Australian Immunisation Handbook recognises Early Childhood Education Centre (ECEC) staff (i.e. those working in childcare, long day care, preschool) as an occupation at increased risk of some vaccine preventable diseases. People who work with children are ‘strongly recommended’ to have measles mumps rubella (MMR) vaccine (if non-immune), pertussis (dTpa), varicella (if non-immune) and hepatitis A. Staff members should also ‘consider’ having the influenza vaccine. Additional vaccinations are recommended for special categories of educators and other staff: hepatitis B for staff who care for children with intellectual disabilities and Japanese encephalitis for those who work in the outer Torres Strait islands for 1 month or more during the wet season. There are currently no mandatory immunisation requirements for Australian ECEC staff members. There is also no supportive funding for vaccines or vaccine administration.

In addition to the Immunisation Handbook, the Australian Government National Health and Medical Research Council published a guideline ‘Staying Healthy: preventing infectious diseases in early childhood education and care services [1]. Aside from reinforcing the immunisation recommendations, it also stresses that ‘all education and care service staff should be advised of the potential consequences if they refuse reasonable requests for immunisation’. These consequences could include: (1) being restricted to working with children over 12 months old; (2) potentially having to take antibiotics during outbreaks of bacterial diseases that are vaccine preventable; and (3) being excluded from work during outbreaks of vaccine-preventable diseases (even if the educator is not ill). It goes on to recommend that ‘employers should develop staff immunisation policy, develop and maintain immunisation records, provide staff with information about vaccine preventable diseases (through in-services training and written material), and take all reasonable steps to encourage non-immune staff to be vaccinated’. Although these guidelines provide for exclusions and encourage vaccination, they are currently not supported by accreditation and licensing requirements.

In 2016, changes were made to the Australian legalisation around the immunisation requirements of children attending kindergarten, long day care, family day care or an occasional care service. Known as the Services Legislation Amendment (No Jab, No Pay/Play) Bill 2015, children need to be fully vaccinated (in accordance with the National Immunisation Program early childhood vaccination schedule, on an approved catch-up schedule or have an approved exemption.) in order to either receive family tax benefits payments and/or in some States/Territories to attend the facility. Vaccine objection (previously known as conscientious objection) is no longer an exemption category. The introduction of the legislation was suggested by some as being coercive because it links the payment of government benefits for childcare to vaccination compliance [2]. Since the introduction of the legislation, the 12 month vaccination coverage has reported to have increased from 92.3 to 93.2% in 2016 [3].

While there is ongoing attention paid to the immunisation of children, there has been little given to the level of coverage amongst staff members. To the best of our knowledge, the last study undertaken on ECEC staff immunization, policy and practice within the Australian child care setting, took place in 2010 [4]. Since this time, the childcare industry has undergone significant growth and regulatory changes. Three notable changes include: (1) the improved educator to child ratio 1:4 (0–2 years) and 1:5 (2–3 years); (2) the requirement for all staff to attain at least a Certificate III qualification and (3) the introduction of the “no jab no pay or play” immunisation strategy (http://www.ncirs.edu.au/consumer-resources/no-jab-no-play-no-jab-no-pay-policies).

Australian childcare services care for an estimated 1.2 million children under 12 [5], with approximately 17,000 public and private operated, government approved childcare centres catering for children aged 6 weeks to 12 years old [5]. These centres provide formal care through long day care (LDC), family day care (FDC), out of school hours (OOSH), vacation care (VC) and to a lesser extent pre-school [6]. To date there has been very few studies conducted looking at the knowledge, attitudes and practices of ECEC staff towards immunisation [7]. This study examined the immunisation status of ECEC staff members as well as their attitudes towards and barriers against occupational vaccination. It also examined their awareness and perceptions towards their employer’s immunisation policies and practices.

## Methods

### Approach

A link to an online questionnaire (using SurveyMonkey) was distributed to ECEC staff members via two approaches. Firstly, an information sheet and link to an anonymous electronic survey was distributed to members of the Australian Childcare Alliance (ACA). The ACA is the national peak body that represents childcare providers across Australia (approximately 2500 members). Secondly, the study was advertised via the Early Childhood Australia (national peak body for early childhood) newsletter and their social media pages (membership numbers unknown). The survey was available online in early 2017. Members of these peak national bodies include those working in a range of ECEC settings: preschool, long day care, family day care, occasional care, mobile services and specialist programs.

The study was approved by the University of New South Wales Human Research Ethics Panel. Consent was implied if the participants completed and submitted the survey,

### Instrument

Questionnaire items were developed using instruments from previous studies examining knowledge, attitudes and practices [8–10]. Additional items were developed which were deemed relevant to the current Australian immunisation situation. The questionnaire contained 59 items plus demographic questions. The questionnaire assessed: (1) attitudes towards awareness of recommendations; (2) perceptions towards risk from infection and staff vaccination; (3) perceived facilitators and barriers to staff vaccination, (4) immunisation status related to pertussis, hepatitis A and B, tetanus and seasonal influenza vaccine; (5) current childcare services policy and practices on staff immunisation; and (6) demographics. The names of the individual diseases were used, rather than the commercial names of the vaccines or the combination vaccines (i.e. dTpa). This may have affected the accuracy of the reporting. In addition, the questionnaire assessed attitudes regarding a proposed staff mandatory vaccination policy. A staff mandatory vaccination policy was defined as a policy requiring all staff except those with a medical contraindication to receive all recommended vaccines to remain employed. Knowledge and attitudes questions were examined by extent of agreement with statements about each vaccine, using a five-point Likert scale (i.e., disagree strongly, disagree somewhat, somewhat agree, and strongly agree, neither agree nor disagree) (Additional file 1). 

### Data analysis

Survey responses using a likert scale were categorized as “expressed agreement” if respondents marked “strongly agree” or “somewhat agree” and as “expressed disagreement” if respondents marked “strongly disagree” or “somewhat disagree” or “neither agree or disagree”. All the categorical variables were summarised as number (%). Characteristics of the childcare centre and their employees were compared between those who reported receipt of each vaccine and those who denied receipt of the respective vaccine using chi-square, or fisher exact and Wilcoxon Signed ranks test wherever appropriate. Vaccine coverage for each vaccine was compared to coverage of influenza vaccines in years 2015 and 2016 using McNemar test for paired data. We compared the responses for the knowledge, attitude and practice questions as well as the questions relating to the centre’s policy and practices between the vaccinated and unvaccinated respondents using Pearson’s Chi Square test. We used a multiple logistic model to identify the factors associated with uptake of different vaccines. We put all the variables that were significantly associated with the specific vaccine uptake based on the bivariate analysis at *p* < 0.20 into the multiple logistic model. However, the variables focused on the centre’s policy and practices had to be removed despite their significant association in the model as they were too many missing values. A backward elimination approached was used based on Akaike Information Criterion to keep the important variables in the final model. We also compared the responses of staff working in privately operated centres with those from other types of centres. All tests were two-tailed, and statistical significance was set at *P* < 0.05. All the analyses were conducted using R3.4.2.

## Results

A total of 576 surveys were commenced and 575 completed. Of the participants, the majority were female (99%), aged 30 years or older (86%) and spoke only English (87%). Around 50% had been in the ECEC workforce for 16 years or more (47%), with a similar number reporting that they worked in a privately-operated centre (50.4%) (Table 1). The survey respondents were representative of the workforce in age and gender, however our survey participants had been in the ECEC workforce longer than average number of years reported in the 2016 ECEC National Workforce Census [11]. Just under half (47.1, 95% CI: 43.0, 51.3) reported receiving an influenza vaccine in 2016. While tetanus uptake was close to 91% (95% CI: 89, 93), uptake for the other vaccines was lower with ranges between 48 and 76% (Table 2). There was no significant difference in the rates of influenza vaccine in 2016 compared to 2015 (*P* = 0.539). The number receiving the hepatitis vaccine was much larger (364/482, 75.5%) compared to those getting influenza vaccine (256/543, 47.1%) in 2016. This difference was of high statistical significance (*P* < 0.001). Similarly, the rates of hepatitis B vaccine were significantly (*P* < 0.001) higher than those receiving the influenza vaccine (260/542, 48%) in 2015. Hepatitis B was significantly (*p* < 0.001) received by a lower number (364/482, 75.5%) of individuals than those receiving tetanus vaccine, which was received by (483/529, 91.3%) participants (Table 2).

**Table 1:** Descriptive statistics of the study sample

Characteristics	Influenza		Pertussis	
	Vaccinated n (%)	Unvaccinated n (%)	Vaccinated n (%)	Unvaccinated n (%)
Sex	*n* = 539		*n* = 527	
Female	250 (46.4)	281 (52.1)	394 (75.9)	125 (24.1)
Male	4 (50)	4 (1.4)	3 (37.5)	5 (62.5)
*p*-value		0.573^*^		0.025^*^
Age (years)	*n* = 543		*n* = 531	
< 30	31 (44.9)	40 (57.9)	58 (89.2)	7 (10.8)
30–39	61 (38.9)	96 (61.1)	125 (79.1)	33 (20.9)
40–49	61 (40.7)	89 (59.3)	107 (71.8)	42 (28.2)
50–59	71 (60.7)	46 (39.3)	73 (65.8)	38 (34.2)
≥ 60	32 (68.1)	15 (31.9)	36 (76.6)	11 (23.4)
*p*-value		0.001		0.020
Country of origin				
Australia	215 (49.8)	217 (50.2)	332 (78.7)	90 (21.3)
England	12 (42.9)	16 (57.1)	20 (74.1)	7 (25.9)
New Zealand	9 (52.9)	8 (47.1)	13 (81.3)	3 (18.7)
Other	20 (30.3)	46 (69.7)	35 (53.0)	31 (47.0)
*p*-value		0.225		< 0.001
Language spoken				
English only	226 (49.3)	232 (50.7)	353 (79.1)	93 (20.9)
Bilingual	22 (33.8)	43 (66.2)	36 (54.5)	30 (45.5)
*p*-value		0.184		< 0.001
Education level				
High school or less	5 (38.5)	8 (61.5)	8 (38.5)	5 (61.5)
Under graduate	127 (52.0)	117 (48.0)	180 (76.3)	56 (23.7)
Postgraduate	28 (39.5)	43 (60.5)	51 (70.8)	21 (29.2)
Certificate/ Diploma	96 (44.7)	119 (55.3)	161 (76.6)	49 (23.4)
*p*-value		0.280		0.365
State				
New South Wales	81 (44.3)	102 (55.7)	147 (80.8)	35 (19.2)
Queensland	61 (50)	61 (50)	105 (83.3)	21 (16.7)
Victoria	59 (44.7)	73 (55.3)	82 (65.1)	44 (34.9)
South Australia	30 (73.2)	11 (27.8)	26 (72.2)	10 (27.8)
Western Australia	11 (34.4)	21 (65.6)	16 (55.2)	13 (44.8)
Other	14 (43.8)	18 (56.2)	24 (77.4)	7 (22.6)
*p*-value		0.031		0.004
Employment status				
Full time	156 (46.7)	178 (53.3)	250 (62.8)	80 (62.5)
Part time	77 (50)	77 (50)	116 (29.1)	36 (28.1)
Casual	17 (40.5)	25 (59.5)	26 (6.5)	10 (7.8)
Student	4 (50)	4 (50)	6 (1.5)	2 (1.6)
*p*-value		0.844		0.545
Child care experience				
< 1 year	6 (30.0)	14 (70.0)	12 (70.6)	5 (29.4)
1–3 years	16 (50)	16 (50)	23 (82.1)	5 (17.9)
4–6 years	27 (49.0)	28 (51.0)	40 (72.7)	15 (27.3)
7–10 years	31 (34.1)	60 (65.9)	66 (75.9)	21 (24.1)
11–15 years	44 (50.6)	43 (49.4)	69 (80.2)	17 (19.8)
≥ 16 years	130 (60.0)	125 (40.0)	189 (74.1)	66 (25.9)
*p*-value		0.130		0.877
Type of day care				
Family day care	17 (53.1)	15 (46.9)	25 (83.3)	5 (16.7)
Long day care- community-run	75 (50.7)	73 (49.3)	106 (71.1)	43 (28.9)
Long day care- privately owned	122 (45.0)	149 (55.0)	211 (79.6)	54 (20.4)
Other	40 (47.6)	44 (52.4)	54 (68.4)	25 (31.6)
*p*-value		0.763		0.090
Centre ownership				
Private	110 (45.8)	130 (54.2)	191 (82.3)	44 (18.7)
Local council	26 (63.4)	15 (36.6)	28 (71.8)	11 (28.2)
Community	31 (47.7)	34 (52.3)	43 (68.3)	20 (31.7)
Employer (University or Company	14 (46.7)	16 (53.3)	24 (80.0)	6 (20.0)
Non-profit organisation	56 (44.1)	71 (55.9)	86 (68.8)	39 (31.2)
Other	15 (46.9)	17 (53.1)	22 (71.0)	9 (29)
*p*-value		0.321		0.029
No of children enrolled				
≤ 20	15 (50.0)	15 (50.0)	18 (64.3)	10 (35.7)
21–50	54 (46.2)	63 (53.8)	83 (70.3)	35 (29.7)
51–100	105 (46.3)	122 (53.7)	169 (77.2)	50 (22.8)
> 100	80 (49.7)	81 (50.3)	126 (79.7)	32 (20.3)
*p*-value		0.720		0.096

^*^ *P* values are derived from Fisher Exact test, for the rest of the variables Wilcoxon Sign rank test was used

**Table 2:** Vaccine coverage among the study participants

Vaccines	Total number of respondents	Number of vaccinated individuals	Vaccine coverage^a^	Comparison of vaccine coverage	*P* value
Influenza (in 2016)	543	256	47.1	Influenza (in 2016) with Influenza (in 2015)	0.539
Influenza (in 2015)	542	260	48.0		
Hepatitis A series^b^	497	365	73.4	Hepatitis A with Influenza (in 2016)	< 0.001
				Hepatitis A with Influenza (in 2015)	< 0.001
Pertussis (Whooping Cough)^c^	531	400	75.3	Pertussis with Influenza (in 2015)	< 0.001
				Pertussis with Influenza (in 2015)	< 0.001
Hepatitis B series^d^	482	364	75.5	Hepatitis B with Influenza (in 2015)	< 0.001
				Hepatitis with Influenza (in 2015)	< 0.001
Tetanus^e^	529	483	91.3	Hepatitis B and tetanus	< 0.001
				Tetanus with Influenza (in 2016)	< 0.001
				Tetanus with Influenza (in 2016)	< 0.001

^a^Coverage has been calculated as (n/N)*100

^b^Two shots ever

^c^In the last 10 years

^d^Three shots ever

^e^Usually provided in combination with diphtheria and pertussis

The responses to the attitudinal questions focused on workplace vaccination policy and practices are presented in Table 3. Not all participants were aware of immunization recommendations outlined in the Australian Immunisation Handbook (67%), though pertussis vaccine recipients were more likely to be aware than the unvaccinated (71% vs 54%, *p* = 0.001). A slightly higher proportion reported being aware of the National Health and Medical Research Council (NHMRC) guidelines, with individuals reporting pertussis vaccination again being more likely to be aware than the unvaccinated individuals (78% vs 62%, *p* < 0.001). Eighty-four percent agreed that ECEC staff should be offered vaccination free of charge, with a significantly higher proportion of the vaccinated individuals agreeing with this statement compared to the unvaccinated individuals (88% vs 70%, *p* < 0.001).

**Table 3:** Comparison of attitudes across child care workers by vaccination status

	Total^a^ Respondents (*N* = 575) n (%)	Pertussis			Influenza		
		Vaccinated (*N* = 400) n (%)	Unvaccinated (*N* = 131) n (%)	*p*-value	Vaccinated (*N* = 256) n (%)	Unvaccinated (*N* = 287) n (%)	*p*-value
Awareness and attitudes towards policy							
I am familiar with the immunisation recommendations for childcare staff in the Australian Immunisation Handbook	381 (66.6)	282 (70.9)	71 (54.2)	0.001	177 (69.4)	178 (62.2)	0.080
I am familiar with the immunisation recommendations for Childcare staff in the NHMRC guidelines	422 (73.6))	313 (78.2)	80 (61.5)	< 0.001	195 (76.5)	201 (70.0)	0.092
Childcare staff can play a role in disease spread if they do not get vaccinated	494 (85.9)	364 (91.0)	91 (69.5)	< 0.001	242 (94.5)	226 (78.7)	< 0.001
Children are required to be vaccinated, therefore staff should be as well	471 (82.1)	345 (86.2)	87 (66.9)	< 0.001	237 (92.6)	206 (72.0)	< 0.001
Vaccination can protect me from illness	498 (87.2)	363 (91.4)	96 (73.3)	< 0.001	247 (97.2)	223 (78.0)	< 0.001
Childcare staff should be offered vaccines free of charge	479 (83.7)	350 (87.9)	91 (69.5)	< 0.001	230 (90.2)	223 (77.7)	< 0.001
Attitudes towards specific infection and vaccine							
Childcare workers have an obligation to be vaccinated against pertussis	486 (84.7)	361 (90.2)	86 (65.6)	< 0.001	NA^b^	NA	
Pertussis can spread from children to childcare staff	505 (88.0)	369 (92.2)	96 (73.3)	< 0.001	NA	NA	
It is important to me to receive the pertussis vaccine	478 (83.3)	370 (92.5)	73 (55.7)	< 0.001	NA	NA	
I would receive the pertussis vaccine, even if I had to pay for it	394 (69.2)	327 (82.0)	45 (34.9)	< 0.001	NA	NA	
I would receive the pertussis vaccine if it were offered to me for free	502 (87.8)	381 (95.2)	84 (64.6)	< 0.001	NA	NA	
I would receive the pertussis vaccine if it were offered to me on-site	476 (83.5)	364 (91.5)	75 (58.1)	< 0.001	NA	NA	
Childcare workers have an obligation to be vaccinated against the flu to reduce the risk of giving the infection to children	355 (61.8)	NA	NA		218 (85.2)	117 (40.8)	< 0.001
Childcare staff should get a flu shot every year unless their doctor tells them they should not	349 (61.1)	NA	NA		223 (87.1)	109 (38.4)	< 0.001
It is important to me to receive the flu vaccine every year	294 (51.5)	NA	NA		224 (87.8)	59 (20.6)	< 0.001
I would receive the flu vaccine every year, even if I had to pay for it	282 (49.1)	NA	NA		217 (84.8)	57 (19.9)	< 0.001
I would receive flu vaccine if it were offered to me free of charge	372 (64.8)	NA	NA		239 (93.4)	118 (41.1)	< 0.001
I would receive the Flu vaccine every year if it were offered to me on-site	375 (65.6)	NA	NA		236 (92.2)	121 (42.5)	< 0.001
ECEC specific approach							
Centre have a specific policy around the vaccination of staff members	197 (43.4)	157 (48.2)	26 (25.7)	< 0.001	86 (43.0)	93 (40.6)	0.617
Centre keep records of the vaccines that staff members have received	289 (59.6)	225 (64.1)	44 (41.9)	< 0.001	135 (60.8)	135 (56.5)	0.346
Employer/supervisor encourage you to get vaccinated	268 (51.3)	204 (56.0)	46 (38.0)	0.001	168 (69.4)	94 (37.0)	< 0.001
Employer/supervisor ask about changes to your vaccination status	167 (31.3)	137 (36.2)	21 (17.4)	< 0.001	82 (33.9)	73 (27.7)	0.129
Centre provide free flu vaccine to staff members	192 (35.7)	NA	NA		108 (44.4)	78 (29.0)	< 0.001
Centre provide onsite flu vaccination	140 (25.5)	NA	NA		88 (34.4)	51 (17.8)	< 0.001

^a^Denominator varies due to missing value

^b^NA, variable not applicable for a specific vaccine

Regarding the introduction of mandatory policies, 69.4% stated that they would support a policy, with moderate increases in support if the vaccine was offered onsite (70.6%) or offered free of charge (75.5%). Only 43% reported that they were aware of a centre specific policy around vaccination, while 60% nominated that their centre keeps records of their vaccination status (Table 3). Of concern was the fact that only 51% reported that their centre encourages them to get vaccinated. Only one-fourth of participants nominated that their centre provided onsite influenza vaccination, with even lower numbers reporting access to free vaccination. Participants were more likely to be fully vaccinated from centres which: have a policy around staff’s vaccination, keep records of their vaccination status, encourage staff to get vaccinated and ask about updates in staff vaccination status. The factors associated with uptake of influenza vaccination: believing that the vaccine protect against illness (Adj. OR: 4.15, 95% CI: 1.44, 12.88), agreeing with the importance of receiving the vaccine each year (Adj. OR: 9.24, 95% CI: 4.16, 21.31) and being happy to pay for it (Adj. OR: 3.13, 95% CI: 1.41, 6.78). Whereas factors associated with hepatitis A vaccine uptake included: agreeing in the importance of the vaccine (Adj. OR: 5.11, 95% CI: 2.04, 13.33), being aged > 30 years, and working in a centre with between 50 and 100 children (Adj. OR: 4.12, 95% CI:1.20, 13.86) (Table 4).

**Table 4:** Factors associated with vaccination against influenza and pertussis

Factors^a^	Pertussis (*n* = 486^b^)		Influenza (*n* = 506)	
	Adj. OR (95% CI)	*p*-value	Adj. OR (95% CI)	*p*-value
Children are required to be vaccinated, therefore staff should be as well	0.48 (0.20, 1.08)	0.086	NIM	
I am familiar with the immunisation recommendations for Childcare staff in the NHMRC guidelines: “*Staying Healthy in ChildCare*”	1.91 (1.03, 3.53)	0.038	NIM	
Vaccination can protect me from illness	NIM^c^		4.15 (1.44, 12.88)	0.010
Childcare staffs should be offered vaccines free of charge	1.98 (0.98, 3.93)	0.053	0.50 (0.21, 1.15)	0.104
It is important to me to receive the flu vaccine every year	NIM		9.24 (4.16, 21.31)	< 0.001
I would receive the flu vaccine every year, even if I had to pay for it	NIM		3.13 (1.41, 6.78)	0.004
I would receive the flu vaccine if it were offered to me free of charge	NIM		2.13 (0.92, 4.99)	0.078
Pertussis can spread from children to childcare staff	1.85 (0.86, 3.91)	0.110	NIM	
It is important to me to receive the pertussis vaccine	3.91 (1.69, 9.38)	0.002	NIM	
I would receive the pertussis vaccine, even if I had to pay for it	6.80 (3.31, 14.20)	< 0.001	NIM	
Age, years				
< 30	1		NIM	
30–39	0.48 (0.16, 1.33)	0.174	NIM	
40–49	0.21 (0.07, 0.57)	0.003	NIM	
50–59	0.13 (0.04, 0.38)	< 0.001	NIM	
≥ 60	0.17 (0.04, 0.64)	0.009	NIM	
Country of origin				
Australia	1			
England	1.98 (0.54, 8.77)	0.334	NIM	
New Zealand	0.73 (0.16, 5.10)	0.719	NIM	
Other	0.51 (0.21, 1.25)	0.134	NIM	
Language Spoken. English	3.07 (1.27, 7.48)	0.013	2.40 (1.10, 5.19)	0.026
State				
NSW	1		NIM	
QLD	1.43 (0.64, 3.26)	0.390	NIM	
VIC	0.57 (0.28, 1.17)	0.127	NIM	
SA	0.37 (0.13, 1.11)	0.066	NIM	
WA	0.28 (0.09, 0.84)	0.021	NIM	
Other	1.00 (0.31, 3.50)	0.994	NIM	
Child care experience				

^a^We did not include the variables on centre’s policy and practice irrespective of their significant association in the bivariate analysis due to missing variables

^b^No of observations used in the multiple logistic model

^c^NIM, Not included in the model

Table 5 presents the differences in knowledge and attitudes amongst participants working in privately owned centres versus other types of centres. Participants from private centres were more likely to be familiar with the immunisation recommendations. One-fourth of participants from privately-owned centres reported that their centre provides flu vaccine free of cost while about half of the staffs from centres owned by others reported the same (*p* < 0.001). About 20% of participants from the privately-owned centres reported that their centre provides onsite flu vaccine while 30% of participants from other types of centres reported onsite access (*p* = 0.013).

**Table 5:** Attitudes towards immunisation policy and practices by participants from privately-owned versus those from other centres

	Total (*N* = 575) n (%)	Privately owned (*N* = 256) n (%)	Community, council/other (*N* = 310) n (%)	*p*-value
I am familiar with the immunisation recommendations for Childcare staff in the Australian Immunisation Handbook	376 (66.5)	188 (73.4)	188 (60.8)	0.002
I am familiar with the immunisation recommendations for Childcare staff in the NHMRC guidelines: “Staying Healthy in Childcare”	418 (73.9)	208 (81.2)	210 (67.7)	< 0.001
The centre provides free flu vaccine to staff members	186 (35.1)	59 (24.4)	127 (44.1)	< 0.001
The centre provides onsite flu vaccine	136 (25.1)	49 (19.8)	87 (29.5)	0.013
The centre encourages the staff to get vaccinated?	262 (50.9)	119 (51.3)	143 (50.5)	0.933
The centre asks about changes to staff member’s vaccination status	166 (31.6)	97 (41.5)	69 (23.6)	< 0.001

## Discussion

Overall, we found mixed levels of awareness regarding the recommendations for vaccination of ECEC staff. Participants reported that not all centres have individual policies regarding staff vaccination, nor do they track uptake. Of concern, was the finding that not all centres are advocating for staff vaccination and very few are providing onsite access. It is perhaps not surprising that we found mixed results when it came to actual vaccine uptake, especially for influenza. Apart from the data presented in this study, there have been few previous attempts to document vaccine uptake in the childcare sector. One study conducted by Spokes et.al over 10 years ago, involved a survey of New South Wales based childcare directors (*n* = 437) to determine their level of knowledge towards the NHMRC recommendations for the immunisation of child-care workers and to ascertain whether the knowledge had translated into practices [12]. The study found that only 49% of the respondents were aware of the policy and just over half had a staff immunisation policy in place. Fast forward to 2017 and while there have been marginal increases in knowledge levels, there has been little in the way of improvements in meeting the NHMRC recommendations. In support of our findings, Spokes et.al also identified that centres that were operating for profit were significantly less likely to offer to pay all or part of the cost of immunisation of staff. Centres that were aware of the NHMRC recommendations and identified as part of a larger organisation were significantly more likely to offer to pay the whole or part of the cost of immunisation. However, this did not remain significant in the multivariate logistic regression [12].

Interestingly, overwhelmingly participants acknowledged that influenza can spread from children to staff member but only 40% agreed that their level of risk was heightened. Encouragingly 60% acknowledge that they felt they had an obligation to be vaccinated against the flu to reduce the risk to the children in their care. However, obligation or willingness to get vaccinated does not always translate to actual receipt with only 50% of participants reported receiving a flu vaccine in 2016. Most of our knowledge about occupational influenza vaccination comes from examining uptake amongst hospital healthcare workers and aged care staff. In those settings, multi-factorial components, such as attitudes, motivation, perceived threat, beliefs, self-efficacy, and socio-cultural influences have all been found to impact on uptake [13].

Inconvenience and cost of vaccination may be having an impact on uptake amongst ECEC staff. There was a positive response to the suggestion of free vaccination amongst participants. To date there has been little work done to examine the impact of free on-site influenza vaccination on childcare staff vaccination prevalence. A small study conducted in one childcare centre over four influenza seasons found that the introduction of free influenza vaccination improved uptake (28 to 51%) [14]. In the healthcare setting, facilities often adopt a range of program strategies to try and improve influenza vaccination uptake amongst healthcare workers. Most sites have attempted to remove these administrative barriers by providing on-site free vaccination services at convenient times that are easily accessible by healthcare workers (HCWs) [15–17]. However, removal of these barriers alone may not necessarily lead to coverage rates above 70%. It has also been suggested in the healthcare setting that too much emphasis has been placed on initiatives like reminders, education, incentives, promotion in the workplace, and easy access to free vaccination, especially considering the small increases in HCW vaccination gained. It has been postulated that more energy needs to be placed on developing interventions which incorporate behavioural psychology and health behaviour change theories [13]. While these recommendations are squarely targeted at healthcare facilities, it could be reasoned that there is value in considering them when developing any interventions aimed at improving uptake in the early childhood education sector.

As previously mentioned, a booster dose of dTpa is recommended for childcare staff if 10 years has elapsed since a previous dose. Given the increased number of pertussis outbreaks documented recently in the United States and elsewhere [18], the finding that one third of participants had not received a booster in the last 10 years is concerning. While there is no evidence to link spread from childcare staff to the children in their care, there has been recent studies suggesting that cases of pertussis are not always linked to household contacts.

For example, a UK study which examined the sources of infection among household contacts of infants under 3 months of age with laboratory-confirmed pertussis, found no identifiable source of infection for half of the cases [19]. The authors proposed that the role of external contacts as source of transmission is often overlooked. Based on data from four countries, they concluded that, in the absence of an identifiable source within the household, around 1 in 3 babies hospitalized were infected by a contact outside the household. Perhaps not surprising that the risk of infection from these sources was suggested to depend on the frequency and intensity of contact [20]. Linking back to the childcare setting, infants (especially those not yet mobile) have frequent and often prolonged close contact with childcare staff members, while they are being comforted, fed and changed. The study authors suggested that beyond routine vaccination of pregnant women, a cocooning strategy (vaccination of contacts of newborns to produce a circle of protection around infants against the disease) that includes other household contacts like fathers, siblings and grandparents should be encouraged [19]. However, there are other important contacts outside that proposed group include healthcare workers, early childhood health nurses, and childcare staff that should be considered.

While the idea of mandatory vaccination for childcare staff is not currently being debated, we felt it was useful to measure the current climate amongst participants towards the idea. We were surprised to see that a large proportion of participants would support mandatory vaccination in the sector. We are not the only ones to document this high level of support. Rebman et.al. also found that most of the parents and staff they surveyed supported a mandatory staff vaccination policy or agency certification program, with no differences in responses between parents versus staff [8]. In support of our findings, Rebman also found that both staff and administrators are more likely to support a mandatory policy if vaccines are offered onsite and free of charge. Only 10% of staff said they would quit if vaccines other than the already-required hepatitis A were mandatory. Further work needs to be undertaken to explore the climate around mandatory vaccination including the support from Australian parents and administrators and the strategies that could be used to govern the introduction.

A major strength of this study is that it is the first to assess nationally current immunisation practices, as well as the level of support for mandatory vaccination policy which could be used in the future to drive strategy change. It is also the first to report the attitudes of childcare staff following the introduction of the new ‘no jab no pay/play’ legalisation. Limitations of this study include the probability of social desirability and selection biases because individuals most interested in staff vaccination were likely to respond. While we received responses from childcare staff across all states/territories, some regional centres may not be appropriately represented. We relied on self-reported vaccine uptake and did not collect data on the uptake of combination vaccines, rather we asked whether participants had received a vaccine against either ‘tetanus’ or ‘pertussis’. This may have led to confusion and potential underestimation of vaccine uptake. Currently, we have no understanding of strategies used within ECEC settings around the documentation, promotion and whether there is any follow up regarding the vaccination of childcare staff. Further work will need to be undertaken to examine whether any activities occur regarding the promotion and/or delivery of immunisation within centres. Lastly, we were unable to accurately calculate a response rate due to the strategies that were used to advertise the study via mailing lists/newsletters.

## Conclusion

ECEC workers may be exposing themselves, the children they care for, colleagues, parents and community members to vaccine preventable diseases because they may not have received the recommended vaccines. This study of immunisation policy and practices in childcare identified deficiencies in awareness towards recommendations, variations in practice around vaccine provision and record keeping and gaps in immunisation uptake. If centres are going to achieve higher coverage rates, then they must reduce the barriers to vaccination, namely through providing free onsite vaccination. Participants were open to idea of mandatory vaccination; however, this needs to be explored further to determine how vaccine costs and access issues could be resolved.

## Additional file

Additional file 1: Survey tool. (DOCX 34 kb)

## Data Availability

De-identified data is available upon request from the corresponding author.

## Abbreviations

ACA: Australian Childcare Alliance
ECEC: Early Childhood Education Centre
FDC: Family day care
HCW: Healthcare workers
LDC: Long day care
NHMRC: National Health and Medical Research Council
OOSH: Out of school hours
VC: Vacation care
