# Supplementary material for: Vaccination uptake among Australian early childhood education staff: assessing perceptions, behaviours and workplace practices
Source: BMC Infect Dis. 2019 Sep 14;19:805. doi: 10.1186/s12879-019-4427-z (PMC6744694; doi:10.1186/s12879-019-4427-z)
Supplement: Supplementary file 1 — Survey tool. (DOCX 34 kb) [file 12879_2019_4427_MOESM1_ESM.docx]

**1. Please rate how much you agree or disagree with each of the following statements**

| Please mark the box that best corresponds to your answer. | Disagree strongly | Disagree somewhat | Neither agree nor disagree | Agree somewhat | Agree strongly |
| --- | --- | --- | --- | --- | --- |
| Childcare staff can play a role in disease spread if they do not get vaccinated | 1 | 2 | 3 | 4 | 5 |
| Childcare workers have an obligation to be vaccinated against **pertussis (Whooping Cough)** to reduce the risk of giving the infection to children | 1 | 2 | 3 | 4 | 5 |
| I am familiar with the immunisation recommendations for Childcare staff in the Australian Immunisation Handbook | 1 | 2 | 3 | 4 | 5 |
| Flu can spread from children to childcare staff | 1 | 2 | 3 | 4 | 5 |
| Children are required to be vaccinated, therefore staff should be as well | 1 | 2 | 3 | 4 | 5 |
| I am familiar with the immunisation recommendations for Childcare staff in the National Health and Medical Research Council guidelines: “*Staying Healthy in ChildCare*” | 1 | 2 | 3 | 4 | 5 |
| Childcare workers have an obligation to be vaccinated against **the flu** to reduce the risk of giving the infection to children | 1 | 2 | 3 | 4 | 5 |
| Pertussis (Whooping Cough) can spread from children to childcare staff | 1 | 2 | 3 | 4 | 5 |
| Vaccination can protect me from illness | 1 | 2 | 3 | 4 | 5 |

**2. Please mark the box that best corresponds to your answer.**

|  | Yes | No | I don’t know |
| --- | --- | --- | --- |
| Does your centre have a specific policy around the vaccination of staff members? | 🞎 | 🞎 | 🞎 |
| Does your centre keep records of the vaccines that staff members have received? | 🞎 | 🞎 | 🞎 |
| Does your centre provide free flu vaccine to staff members? | 🞎 | 🞎 | 🞎 |
| Does your employer/supervisor recommend you get vaccinated? | 🞎 | 🞎 | 🞎 |
| Does your centre provide onsite flu vaccination? | 🞎 | 🞎 | 🞎 |
| Does your employer have a policy that encourages you to stay at home when you are sick? | 🞎 | 🞎 | 🞎 |
| Does your employer/supervisor encourage you to get vaccinated? | 🞎 | 🞎 | 🞎 |
| Does your employer/supervisor ask about changes to your vaccination status? | 🞎 | 🞎 | 🞎 |

**3. Please rate how much you agree or disagree with each of the following statements**

| Please mark the box that best corresponds to your answer. | Disagree strongly | Disagree somewhat | Neither agree nor disagree | Agree somewhat | Agree strongly |
| --- | --- | --- | --- | --- | --- |
| It is important to me to receive the **pertussis (Whooping Cough) vaccine** | 1 | 2 | 3 | 4 | 5 |
| I would receive the **pertussis (Whooping Cough) vaccine**, even if I had to pay for it | 1 | 2 | 3 | 4 | 5 |
| I would receive the **pertussis (Whooping Cough) vaccine** if it were offered to me **for free** | 1 | 2 | 3 | 4 | 5 |
| I would receive the **pertussis (Whooping Cough) vaccine** if it were offered to me **on-site** |  |  |  |  |  |
| Childcare staff should get a **flu** shot every year unless their doctor tells them they shouldn’t | 1 | 2 | 3 | 4 | 5 |
| It is important to me to receive the **flu vaccine** every year | 1 | 2 | 3 | 4 | 5 |
| I would receive the **flu vaccine** every year, even if I had to pay for it | 1 | 2 | 3 | 4 | 5 |
| I would receive **flu vaccine** if it were offered to me **free of charge** | 1 | 2 | 3 | 4 | 5 |
| I would receive the **Flu vaccine** every year if it were offered to me **on-site** | 1 | 2 | 3 | 4 | 5 |
| Childcare staff should be offered vaccines free of charge | 1 | 2 | 3 | 4 | 5 |
| It is important to me to receive the **Hepatitis A vaccine** | 1 | 2 | 3 | 4 | 5 |
| I would receive the **Hepatitis A vaccine**, even if I had to pay for it | 1 | 2 | 3 | 4 | 5 |
| I would receive the **Hepatitis A vaccine** if it were offered **free of charge** | 1 | 2 | 3 | 4 | 5 |
| I would receive the **Hepatitis A vaccine** if it were offered to me **on-site** | 1 | 2 | 3 | 4 | 5 |

**4. Which of the following vaccine(s) have you received?**

|  | **Yes** | **No** | **I don’t know** |
| --- | --- | --- | --- |
| Influenza (in 2016) | 🞎 | 🞎 | 🞎 |
| Influenza (in 2015) | 🞎 | 🞎 | 🞎 |
| Hepatitis A series (2 shots needed) (ever) | 🞎 | 🞎 | 🞎 |
| Pertussis (Whooping Cough) (in the last 10 years) | 🞎 | 🞎 | 🞎 |
| Hepatitis B series (3 shots needed) (ever) | 🞎 | 🞎 | 🞎 |
| Tetanus (usually in combination with diphtheria and pertussis i.e. DTPa) | 🞎 | 🞎 | 🞎 |

**5. Do you plan to receive the flu vaccine in 2017?**

☐ No

☐ Yes

☐ Have not decided

**6.** **What percentage of your colleagues do you think received the flu vaccine in 2016? ________%**

**7. The following are possible reasons why someone might or might not decide to get vaccinated. Please rate how much you agree or disagree with each of the following statements**

| **Please mark the box that best corresponds to your agreement with each statement.** | Disagree strongly | Disagree somewhat | Neither agree nor disagree | Agree somewhat | Agree strongly |
| --- | --- | --- | --- | --- | --- |
| Vaccination can protect me from illness | 1 | 2 | 3 | 4 | 5 |
| Vaccines have a lot of side effects | 1 | 2 | 3 | 4 | 5 |
| I believe vaccines are safe | 1 | 2 | 3 | 4 | 5 |
| My immune system has become built up from years of being around children, so I do not think it is likely that I will get sick | 1 | 2 | 3 | 4 | 5 |
| I was vaccinated for **pertussis (Whooping Cough)** when I was a child, so I don’t need the **pertussis vaccine** | 1 | 2 | 3 | 4 | 5 |
| **Pertussis (Whooping Cough)** doesn’t occur in Australia anymore, so I don’t need the **pertussis vaccine** | 1 | 2 | 3 | 4 | 5 |
| I am at risk for getting **Hepatitis A** | 1 | 2 | 3 | 4 | 5 |
| I am afraid of potential side effects from **flu vaccine** | 1 | 2 | 3 | 4 | 5 |
| I could get the flu from the **flu vaccine** | 1 | 2 | 3 | 4 | 5 |
| Without the **flu vaccine**, I am more likely to get the **flu** than other people | 1 | 2 | 3 | 4 | 5 |
| It is too inconvenient for me to get the **flu vaccine** | 1 | 2 | 3 | 4 | 5 |
| **Flu vaccine** is only for older individuals | 1 | 2 | 3 | 4 | 5 |
| I was vaccinated for flu in the past, so I don’t need **flu vaccine** again | 1 | 2 | 3 | 4 | 5 |
| My employer believes that it is important that staff members are vaccinated | 1 | 2 | 3 | 4 | 5 |
| My colleagues believe that it is important that staff members are vaccinated | 1 | 2 | 3 | 4 | 5 |

**8. Some people believe that a “mandatory vaccination” policy should be in place for childcare staff, meaning that all staff (except those who cannot get vaccinated for medical reasons) would need to receive all vaccines recommended in order to stay employed. The following set of questions relate to your opinion about a *mandatory vaccination policy* for childcare agency staff.**

| Please mark the box that best corresponds to your answer. | Disagree strongly | Disagree somewhat | Neither agree nor disagree | Agree somewhat | Agree strongly |
| --- | --- | --- | --- | --- | --- |
| I would support a mandatory vaccination policy for childcare staff | 1 | 2 | 3 | 4 | 5 |
| My support for a mandatory policy depends on whether I had to cover some or all of the cost | 1 | 2 | 3 | 4 | 5 |
| I would support a mandatory vaccination policy if the vaccines were offered **on-site** | 1 | 2 | 3 | 4 | 5 |
| I would support a mandatory vaccination policy if the vaccines were offered **free of charge** | 1 | 2 | 3 | 4 | 5 |
| I would support a mandatory vaccination policy if my clients’ parents wanted such a policy | 1 | 2 | 3 | 4 | 5 |

**9. Demographics**

**What is your gender?** □ Female □ Male

**What is your age?** ☐ ≤ 20 ☐ 21 – 30 ☐ 31 – 40 ☐ 41 – 50 ☐ 51 – 60 ☐ ≥ 61

**What is your country of birth**

□ Australia

□ England

□ New Zealand

□ India

□ China

□ Vietnam

□ Philippines

□ Other, please *specify*_____________

**Do you speak a language other than English?**

□ No, English only

□ Yes, Mandarin

□ Yes, Italian

□ Yes, Arabic

□ Yes, Cantonese

□ Yes, Vietnamese

□ Yes, other, please *specify*_____________

**What is the level of the highest qualification that you have completed (i.e. Bachelor degree, certificate 11)__________________________________________**

or

□ I am still studying for my first qualification

**What State/Territory do you work in?**

□ ACT □ NSW □ NT □ QLD □ SA □ TAS □ VIC □ WA

**What is your work status?** ☐ Part-time or as needed ☐ Full-time

**For how many years have you been a childcare provider?** ☐ < 1 ☐ 1 – 2 ☐ 3 – 4 ☐ 5 – 10 ☐ ≥ 11

**What category describes the centre that you work at?**

□ Long day care □ Pre-school □ other, please specify________________________

**Who operates the centre?**

□ Private operator □ Local council □ Community organisation □ Employer (i.e. university or company) □ Non-profit organisation

**How many employees does your centre have?** ☐ ≤ 5 ☐ 6 - 10 ☐ 11 - 25 ☐ ≥ 26

**Is the centre?** □ A single centre □ Part of a chain

**How many children are currently enrolled in your centre?** ☐ ≤ 5 ☐ 6-10 ☐ 11-25 ☐ 26-50 ☐ 51 - 99 ☐ ≥100

**What is the age of the children for whom you provide care most often?**

☐ 0 - 5 months ☐ 6 - 12 months ☐ 13 months – 3 years ☐ 4 years or older

☐ Not applicable; I only perform administrative duties
